# Supplementary material for: Transposable elements potentiate radiotherapy-induced cellular immune reactions via RIG-I-mediated virus-sensing pathways
Source: Commun Biol. 2023 Aug 5;6:818. doi: 10.1038/s42003-023-05080-x (PMC10404237; doi:10.1038/s42003-023-05080-x)
Supplement: Supplementary file 2 — Supplementary information [file 42003_2023_5080_MOESM2_ESM.pdf]

Supplementary Fig. 1

a

Correlation of expression in NSCLC tissues

(Spearman's Correlation)

	RIG-I	MDA5	cGAS	IFNG
OAS2	0.699	0.744	0.274	0.257
MX1	0.651	0.662	0.162	0.06
OASL	0.499	0.578	0.345	0.359
IFNB1	0.304	0.294	0.305	0.153
IRF7	0.322	0.342	0.092	0.084
IRF9	0.539	0.471	0.156	0.259

b

Correlation of RIG-I or MDA-5 expression with cell type profiles

(Spearman's p)

	RIG-I	MDA5
Macrophage_TIMER	1.70E-05	0.863343
Myeloid dendritic cell_TIMER	2.43E-07	0.000221
Neutrophil_TIMER	2.19E-26	0.436475
B cell_TIMER	0.15761	4.19E-05
T cell CD4+_TIMER	0.074704	6.17E-09
T cell CD8+_TIMER	7.94E-17	0.506336

c

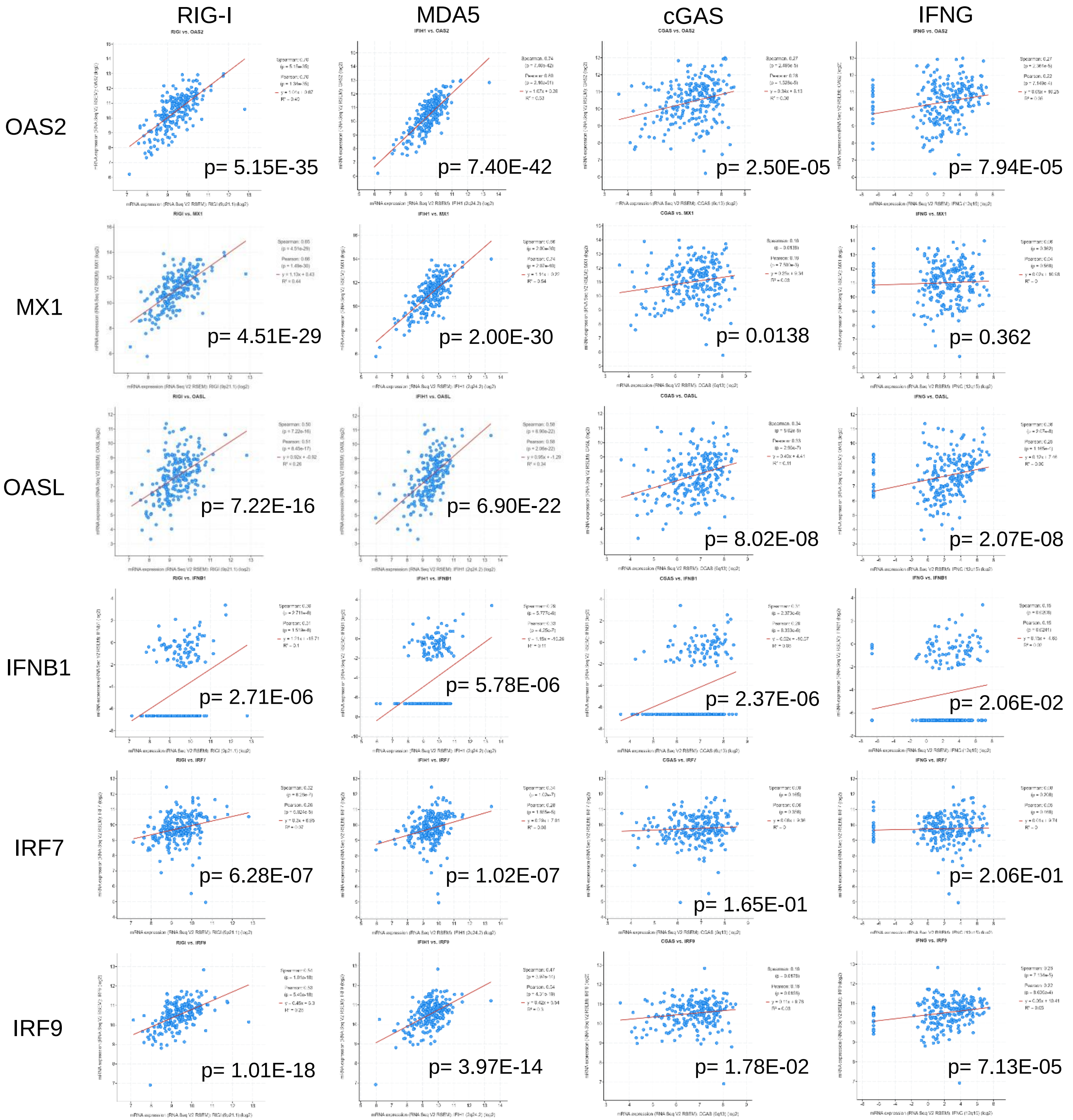

Supplementary Fig. 1

a. Spearman's correlation analysis of expression levels in a TGCA lung adenocarcinoma dataset (Firehose Legacy, n=584).

b-c. Correlation(b) and plot(c) of RIG-I or MDA-5 expression with cell type profiles calculated using TIMER2.0 web tools (http://timer.comp-genomics.org/).

Supplementary Fig. 2

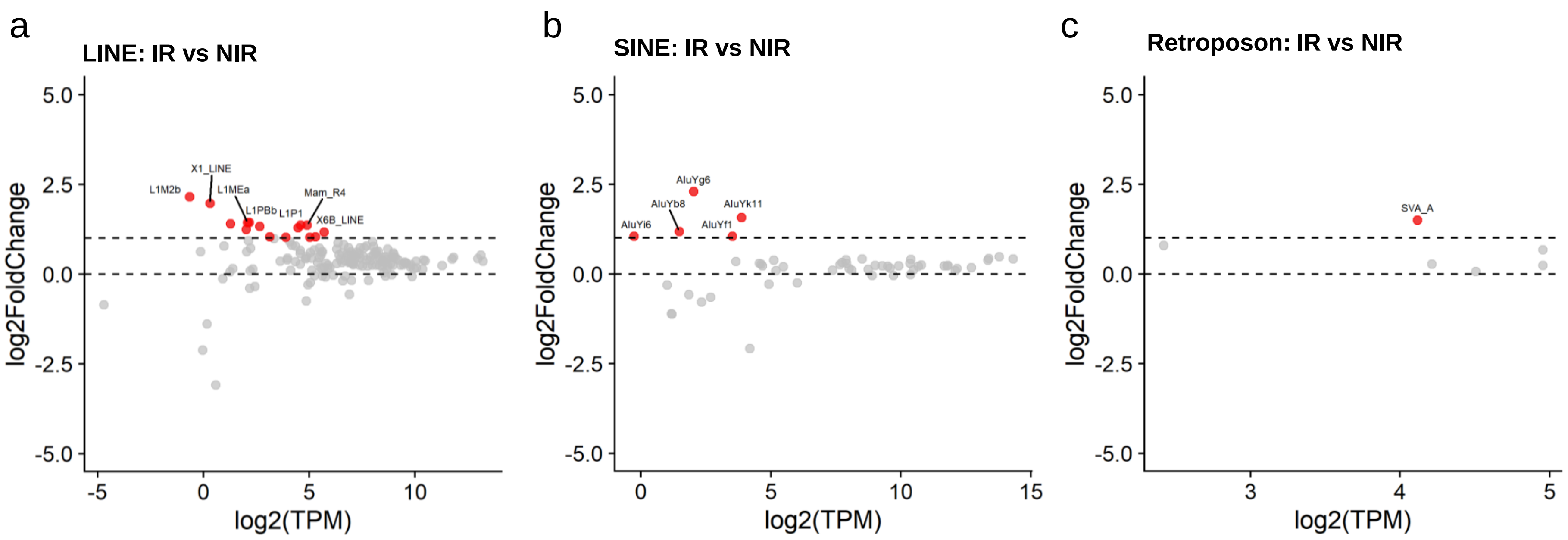

Supplementary Fig. 2

a-c. Fold changes in LINE (**a**), SINE (**b**), and retroposon (**c**) expression between irradiated (IR) and nonirradiated (NIR) samples. The IR group was harvested 7 days after 8 Gy irradiation, and the NIR group was harvested at the same time without irradiation. The expression of the indicated TEs was quantified by total RNA-seq. Data are inclusive of two independent experiments. The red dots indicate TEs with more than a 2-fold change in abundance in IR cells compared with NIR cells.

Supplementary Fig. 3

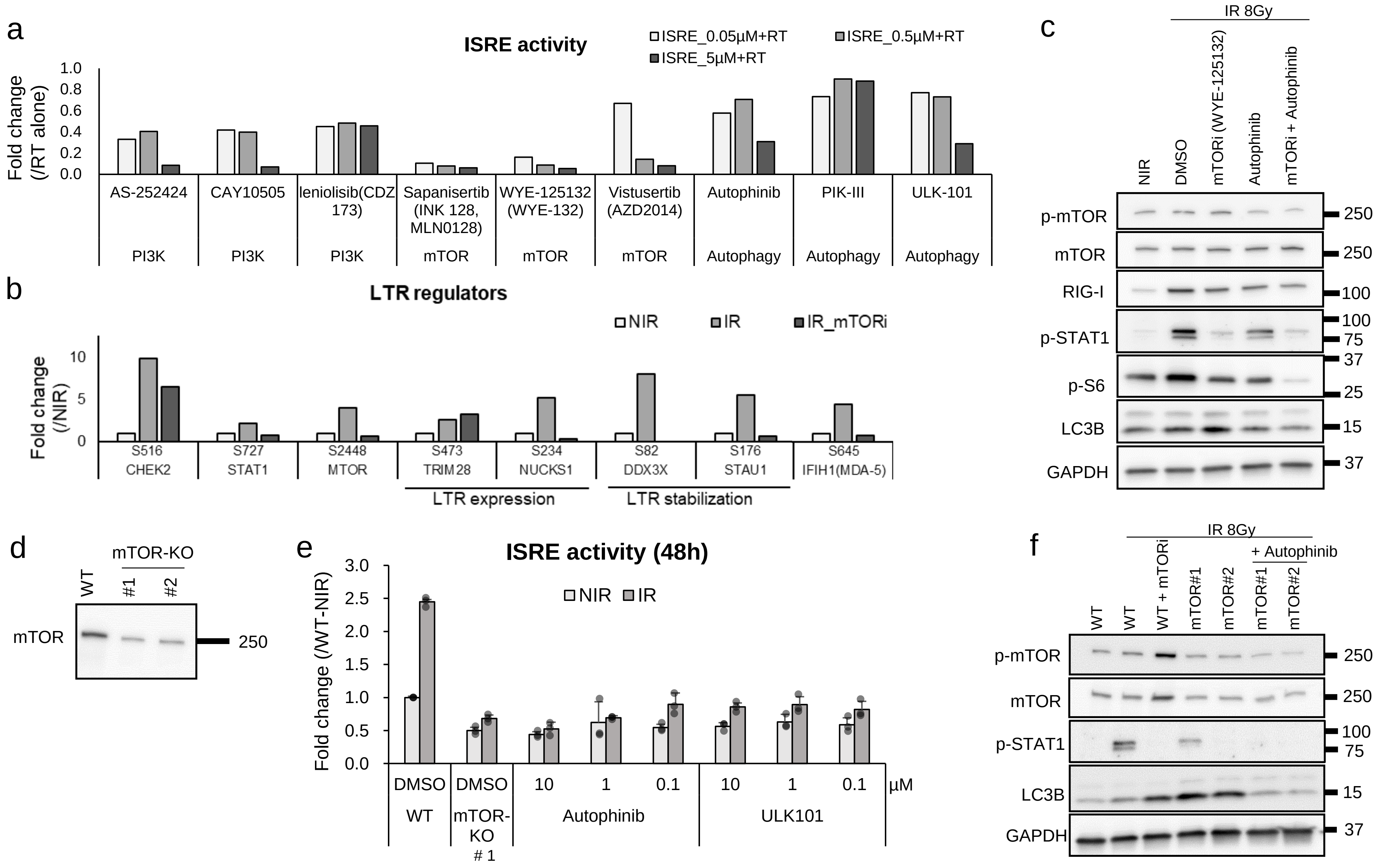

Supplementary Fig. 3

a. ISRE activity in A549 cells after treatment for 96 h with RT and the indicated inhibitors at the indicated concentrations. Data are shown as the fold change relative to RT alone.

b. Phosphorylation of representative LTR regulators in NIR cells and IR cells treated with or without an mTOR inhibitor (WYE-125132, 50 nM). Data are shown as the fold change relative to NIR.

c. Total and phosphorylated levels of representative mTOR pathway components and autophagy-related proteins in nonirradiated (NIR) cells and irradiated (IR) cells treated with DMSO, the mTORi (WYE-125132, 100 nM), the autophagy inhibitor autophinib (1 μM), or mTORi + autophinib.

d. Western blot of mTOR protein levels in wild-type (WT) A549 cells and two clones with genetic knockout (KO) of mTOR.

e. Fold change in ISRE activity in WT and mTOR-KO A549 cells at 48 h after the indicated treatment.

f. Total and phosphorylated protein levels of mTOR and markers of immune activation in A549 cells treated as indicated.

Supplementary Fig. 4

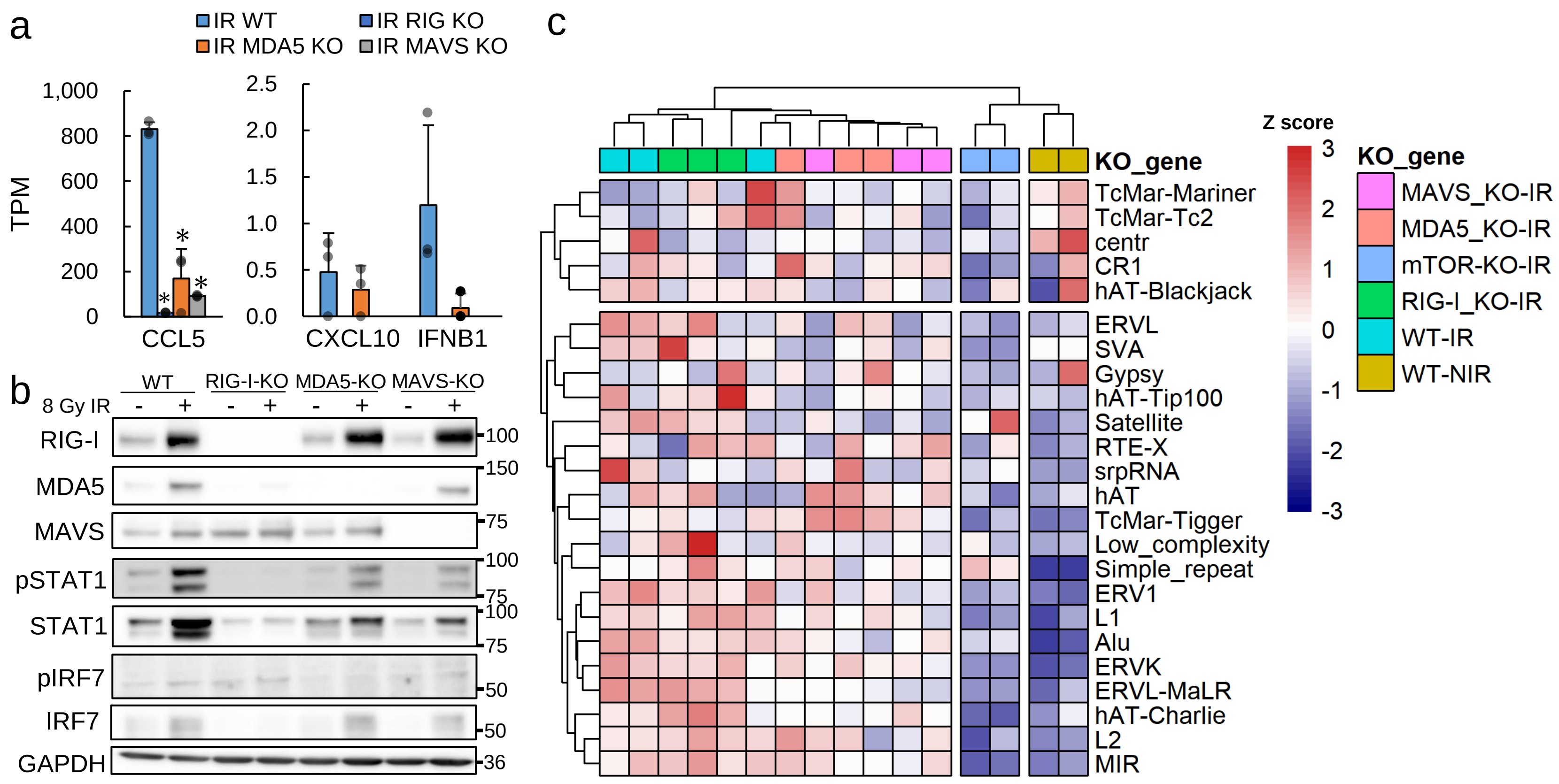

Supplementary Fig. 4

a. Gene expression of representative markers of immune activation assessed by RNA-seq in irradiated (IR) WT, RIG-I knockout (KO), MDA5-KO, and MAVS-KO cells. CSCL10 and IFNB1 were not detected in IR RIG-I-KO or IR MAVS-KO cells. Error bars represent the SEM of 3 biological replicates. *$p \leq 0.05$ compared to the IR WT group, Student's t test.

b. Protein expression of representative immune-active markers in the cells specified in (**a**) treated with or without 8 Gy irradiation.

c. Expression of TEs assessed by total RNA-seq in nonirradiated (NIR) WT cells and irradiated (IR) WT, mTOR-KO, RIG-I-KO, MDA5-KO, and MAVS-KO cells. The Z score was calculated from the TPM of the indicated TEs, and clustering was performed using the R package pheatmap.

Supplementary Fig. 5

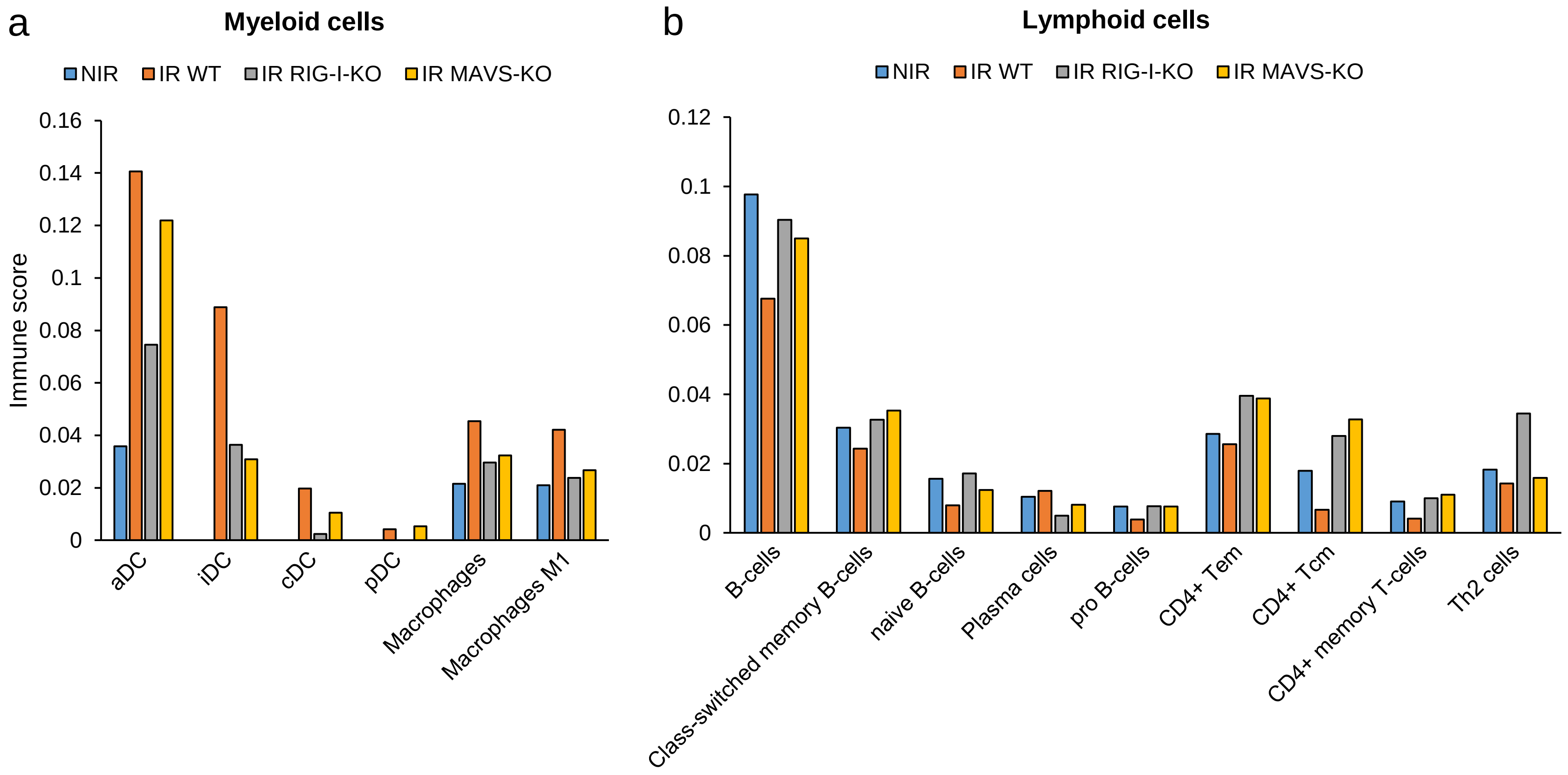

Supplementary Fig. 5

a-b. Myeloid cell (**a**) and lymphoid cell (**b**) immune scores of PBMCs cultured with medium from the indicated cells. The immune scores were calculated by xCell.

Supplementary Fig. 6

a

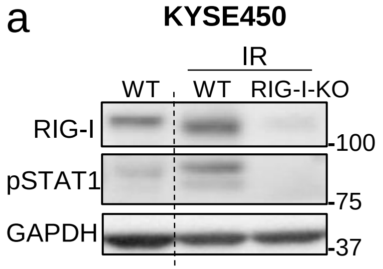

Supplementary Fig. 6

Protein levels of RIG-I and the immune-activated marker pSTAT1 in the WT and RIG-KO KYSE-450 oesophageal cancer cell lines after 15 Gy irradiation, with GAPDH as a loading control.

Supplementary Fig. 7

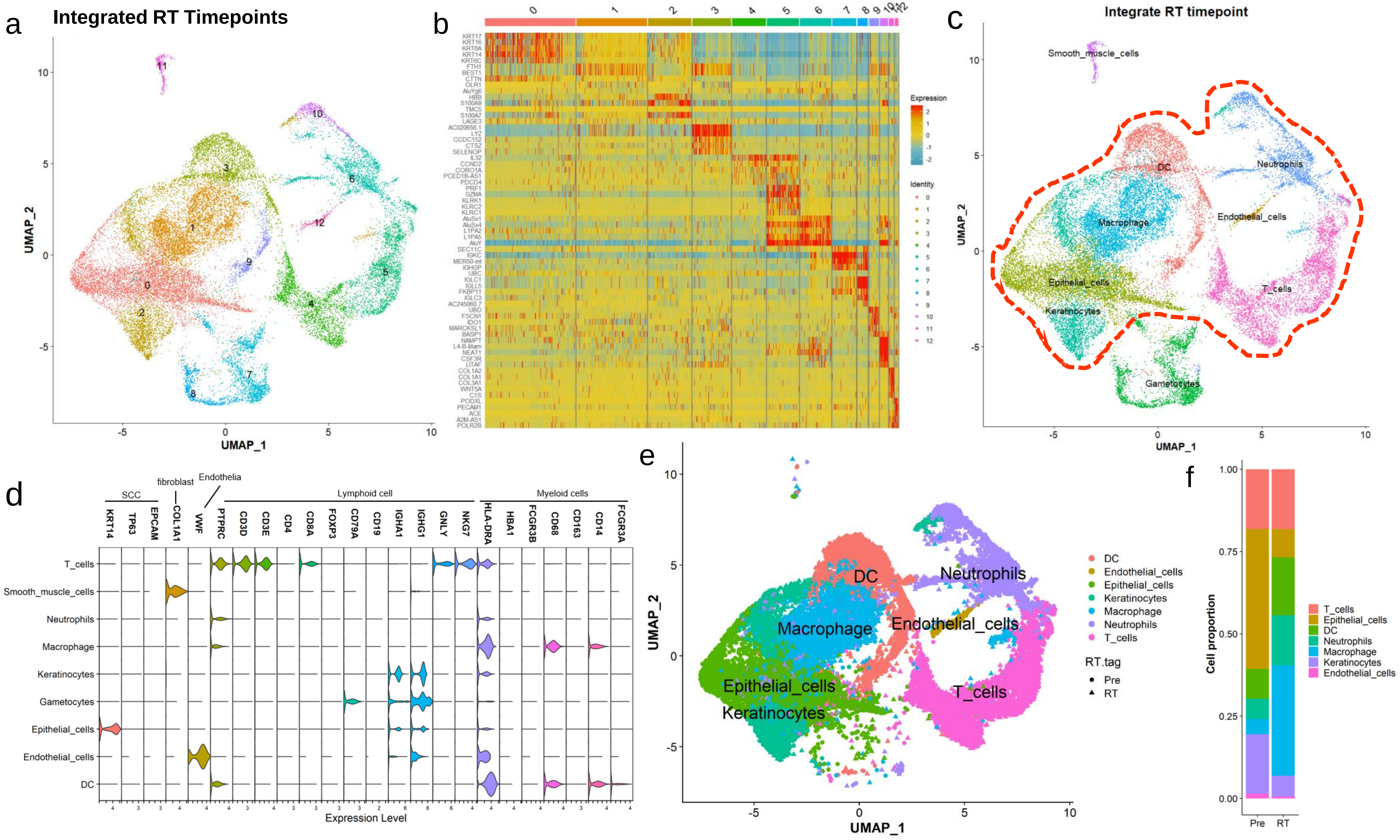

Supplementary Fig. 7

a. UMAP plot of the integrated scRNA-seq object.

b. Identification of characteristic marker genes for each cluster in **(a)**.

c. SingleR annotation of the integrated scRNA-seq object. The red dashed line encompasses the cell clusters selected for comparative analysis.

d. Violin plot of the expression of representative marker genes in each cell cluster annotated in (**c**).

e. UMAP plot of the cell clusters selected for comparative analysis.

f. Proportions of the cell clusters annotated in (**e**).

Supplementary Fig. 8

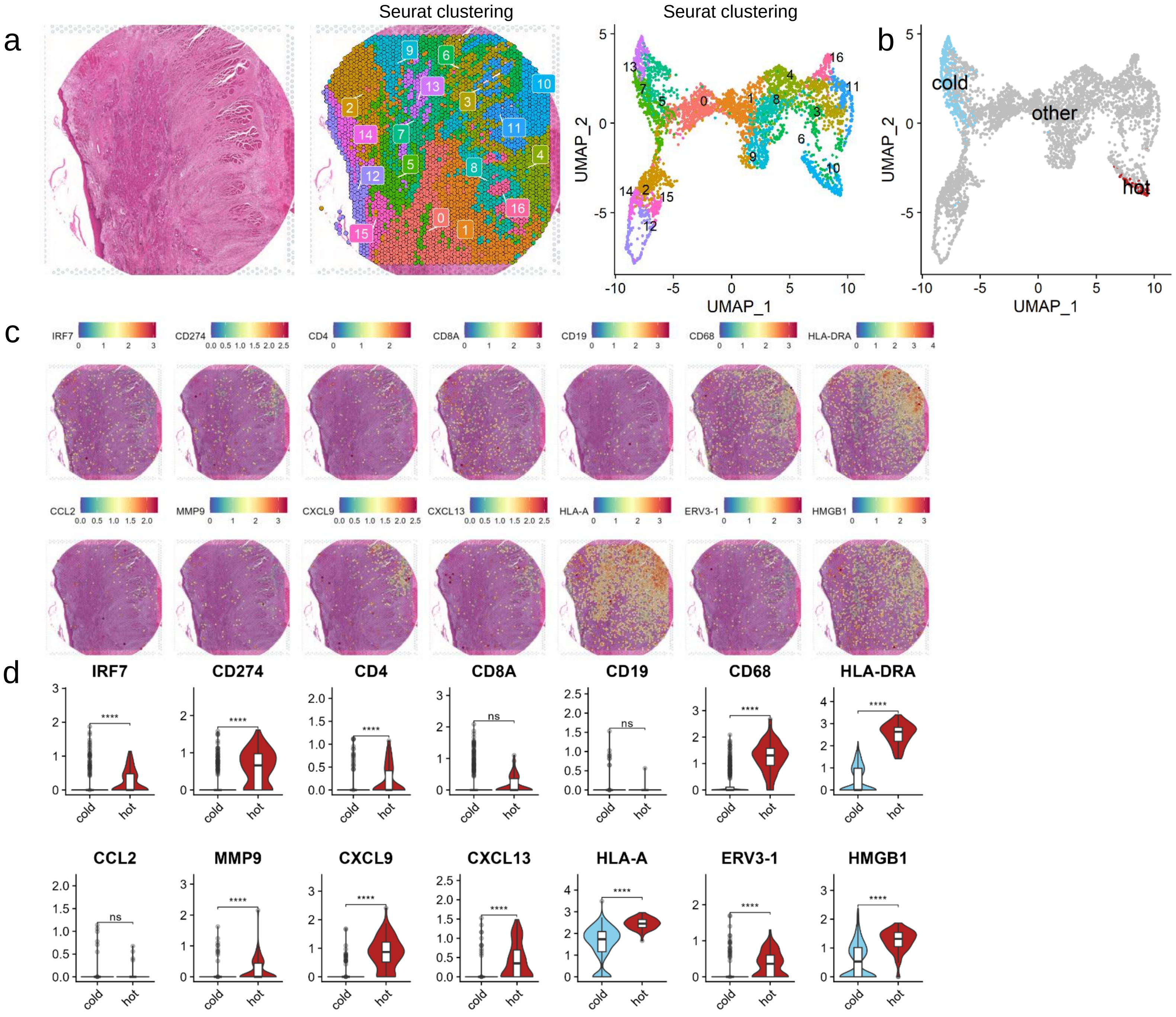

Supplementary Fig. 8

a. Morphology of the 10x Visium slide (left), Seurat clusters of spots according to morphology image (middle), and UMAP plot of the Seurat clusters (right).

b. Manual annotation of hot and cold sites according to the UMAP clustering of the spots. Hot sites contain damaged cancer cells and considerable immune cell infiltration, and cold sites contain dense, viable cancer cells and little immune cell infiltration.

c. Spatial expression of representative cell markers in the spots.

d. Quantitation of the expression of representative cell markers in the spots shown in (**b**). ****$p < 1 \times 10^{-16}$, Bonferroni-adjusted Wilcoxon test; ns, no significance.

Supplementary Fig. 9

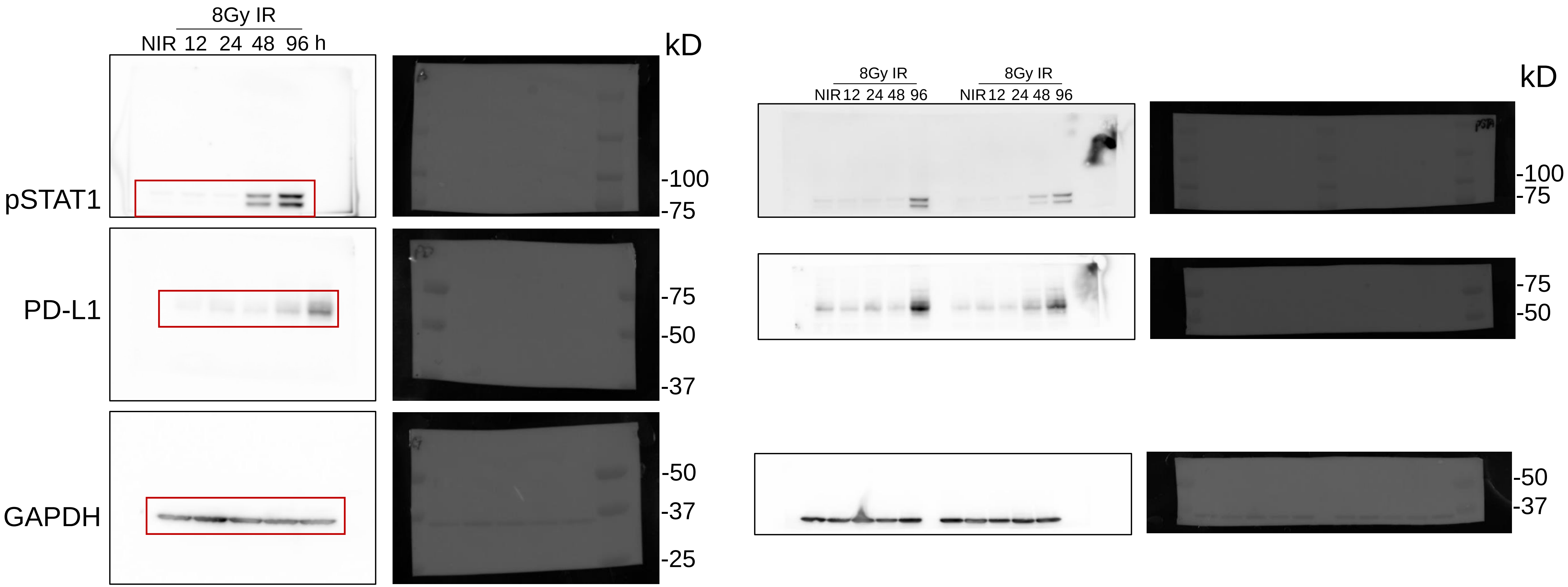

Supplementary Fig. 10

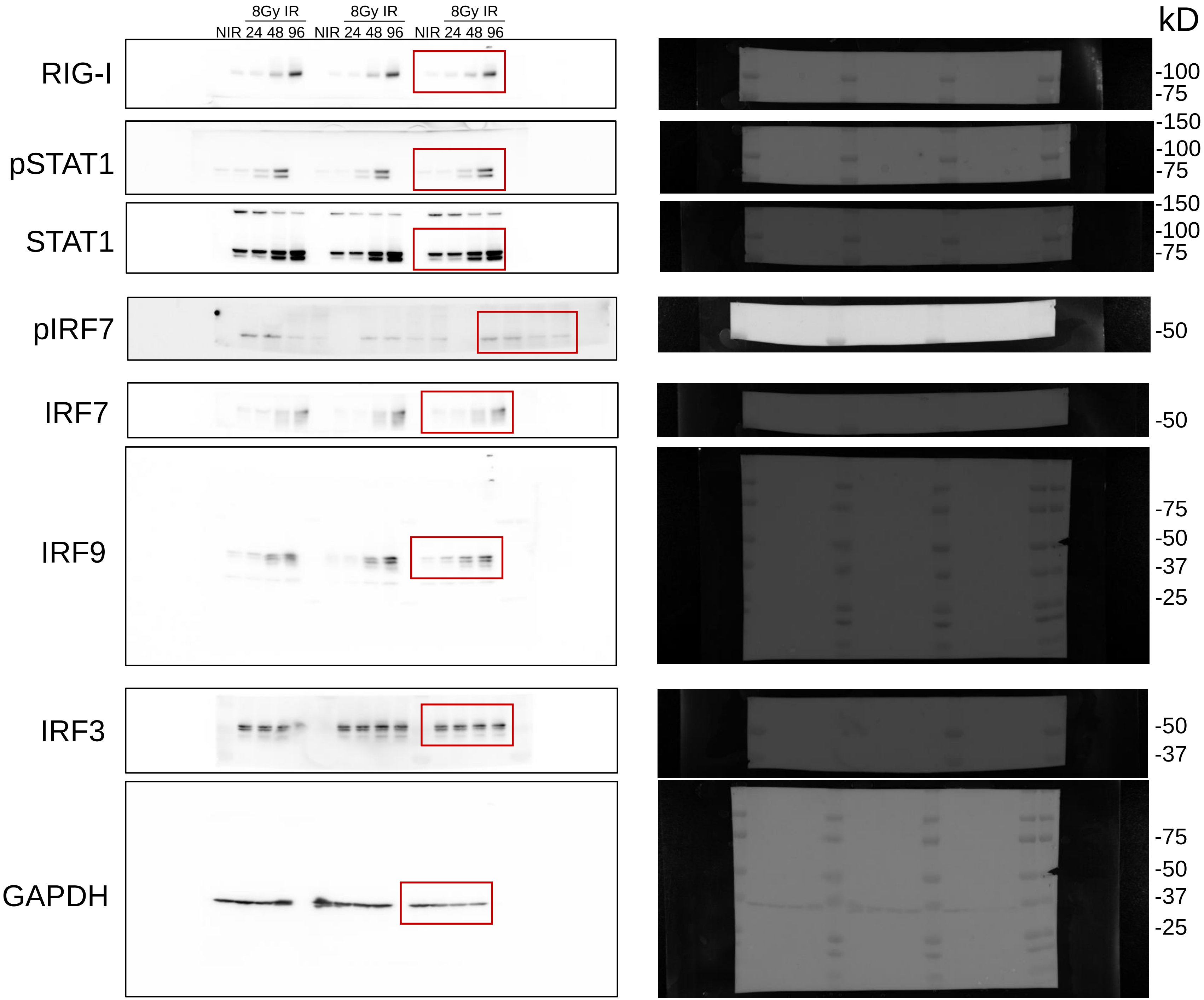

Supplementary Table 1

Patient character for scRNA seq and spatial transcriptome

Number	Gender	Age, years	Diagnosis	sStage (UICC 7th)	Pre RT	After RT	Analysis
Case 1	M	78	ESCC	Ut, ESCC T4N1M0 cStage IIIC	Case 1-1 (pre)	Case 1-2 (24Gy/12fr)	scRNA seq
Case 2	M	57	ESCC	Mt, ESCC T4bN2M0 cStage IVa	/	Case 2-2 (60Gy/30fr)	scRNA seq
Case 3	M	90	ESCC	Lt, ESCC T4aN1M0 cStage IIIB	Case 3-1 (pre)	/	scRNA seq
Case 4	M	73	ESCC	Ce, ESCC T4bN2M1(#104R) cStage IVb	/	Case 4-2 (25.2Gy/14fr)	scRNA seq
Case 5	M	58	ESCC	cT3N3M0 cStage IIIC	/	Case 5-2 (41.2Gy/23fr)	Spatial transcriptome
