# Supplementary material for: Transposable elements potentiate radiotherapy-induced cellular immune reactions via RIG-I-mediated virus-sensing pathways
Source: Commun Biol. 2023 Aug 5;6:818. doi: 10.1038/s42003-023-05080-x (PMC10404237; doi:10.1038/s42003-023-05080-x)
Supplement: Supplementary file 5 — Reporting Summary [file 42003_2023_5080_MOESM5_ESM.pdf]

nature portfolio

Corresponding author(s): Shun-ichiro Kageyama

Last updated by author(s): Jun 5, 2023

Reporting Summary

Nature Portfolio wishes to improve the reproducibility of the work that we publish. This form provides structure for consistency and transparency in reporting. For further information on Nature Portfolio policies, see our Editorial Policies and the Editorial Policy Checklist.

Statistics

For all statistical analyses, confirm that the following items are present in the figure legend, table legend, main text, or Methods section.

n/a	Confirmed	
☐	☒	The exact sample size (*n*) for each experimental group/condition, given as a discrete number and unit of measurement
☐	☒	A statement on whether measurements were taken from distinct samples or whether the same sample was measured repeatedly
☐	☒	The statistical test(s) used AND whether they are one- or two-sided<br>*Only common tests should be described solely by name; describe more complex techniques in the Methods section.*
☒	☐	A description of all covariates tested
☒	☐	A description of any assumptions or corrections, such as tests of normality and adjustment for multiple comparisons
☒	☐	A full description of the statistical parameters including central tendency (e.g. means) or other basic estimates (e.g. regression coefficient) AND variation (e.g. standard deviation) or associated estimates of uncertainty (e.g. confidence intervals)
☒	☐	For null hypothesis testing, the test statistic (e.g. *F*, *t*, *r*) with confidence intervals, effect sizes, degrees of freedom and *P* value noted<br>*Give P values as exact values whenever suitable.*
☒	☐	For Bayesian analysis, information on the choice of priors and Markov chain Monte Carlo settings
☒	☐	For hierarchical and complex designs, identification of the appropriate level for tests and full reporting of outcomes
☐	☒	Estimates of effect sizes (e.g. Cohen's *d*, Pearson's *r*), indicating how they were calculated

Our web collection on statistics for biologists contains articles on many of the points above.

Software and code

Policy information about availability of computer code

Data collection	No software were used.
Data analysis	RNA purification and bioinformatic analysis<br>For RIP RNA-seq, total RNA-seq was performed by Macrogen (Tokyo, Japan) with a SMARTer stranded kit (Clontech Laboratories). Sequencing data were subjected to quality control filtering, trimming, and adaptor removal using FastQC and Trimmomatic. All filtered sequences were aligned to the hg38 reference genome, and gene expression was represented as transcripts per million (TPM) calculated by RSEM. Differentially expressed genes (DEGs) were identified by using the R package edgeR51 with a false discovery rate (FDR) ≤0.05. DEGs were used for Gene Ontology (GO) and Kyoto Encyclopedia of Genes and Genomes (KEGG) analyses with the R package clusterProfiler. TE expression analysis was performed using the REdiscoverTE pipeline. Heatmaps were drawn using the R package pheatmap.<br><br>Single-cell RNA sequencing (scRNA-seq)<br>Cell Ranger (ver. 3.0.0) was used with default parameters to process the reference genome alignment and quantify cells and transcripts. The raw sequencing reads were mapped to the human genome assembly GRCh38. TE levels based on the scRNA-seq data were further quantified with the scTE pipeline using the BAM files generated by Cell Ranger. The single-cell expression data generated by scTE were further analysed with the R package SeuratV4. Cells with a gene number > 5000 or < 200 or a mitochondrial gene ratio > 10% were excluded from downstream analysis. Five scRNA-seq samples were collected, and the data were integrated using the sctransform pipeline in Seurat. The integrated object was then normalized and scaled. Next, principal component analysis (PCA) (RunPCA) was performed. The top 30 principal components (PCs) were selected and submitted to FindNeighbors, FindClusters, and UMAP (RunUMAP) to obtain clusters. Cell type was annotated manually by referring to published papers and by SingleR (ver. 1.6.1). The annotated cell clusters were verified using a known cell marker list. Immune cell clusters (macrophages, DCs, neutrophils, T cells, and B cells) and epithelial cells were selected for comparative analysis.

10x Visium spatial transcriptomic analysis
The Space Ranger pipeline was used to process the Visium spatial gene expression data, including data generated from demultiplexing, hg38 human reference genome alignment, tissue detection, fiducial detection, and barcode/UMI counting, following the guidelines provided by the manufacturer.

For manuscripts utilizing custom algorithms or software that are central to the research but not yet described in published literature, software must be made available to editors and reviewers. We strongly encourage code deposition in a community repository (e.g. GitHub). See the Nature Portfolio guidelines for submitting code & software for further information.

Data

Policy information about availability of data

All manuscripts must include a data availability statement. This statement should provide the following information, where applicable:

- Accession codes, unique identifiers, or web links for publicly available datasets
- A description of any restrictions on data availability
- For clinical datasets or third party data, please ensure that the statement adheres to our policy

Data is provided on public data repository or as Supplementary Data.

Human research participants

Policy information about studies involving human research participants and Sex and Gender in Research.

Reporting on sex and gender	Sex and gender was not considered in the study.
Population characteristics	Population characteristics were provided in the Supplementary table.2.
Recruitment	Tissue donors were recruited by collaborating doctors in the National Cancer Center Japan. There is no potential self-selection in the volunteer tissue donation process.
Ethics oversight	All analyses were performed with patient consent and IRB approval from the National Cancer Center East Hospital (IRB2018-101).

Note that full information on the approval of the study protocol must also be provided in the manuscript.

Field-specific reporting

Please select the one below that is the best fit for your research. If you are not sure, read the appropriate sections before making your selection.

☒ Life sciences ☐ Behavioural & social sciences ☐ Ecological, evolutionary & environmental sciences

For a reference copy of the document with all sections, see nature.com/documents/nr-reporting-summary-flat.pdf

Life sciences study design

All studies must disclose on these points even when the disclosure is negative.

Sample size	The sample size was not predetermined and all samples that were available were processed.
Data exclusions	scRNA-seq analysis: Cells with a gene number > 5000 or < 200 or a mitochondrial gene ratio > 10% were excluded from downstream analysis.
Replication	2-4 replications were done except for RIP-seq and human samples due to practical constraints.
Randomization	Different single cells were randomly captured before analysis.
Blinding	Not relevant.

Reporting for specific materials, systems and methods

We require information from authors about some types of materials, experimental systems and methods used in many studies. Here, indicate whether each material, system or method listed is relevant to your study. If you are not sure if a list item applies to your research, read the appropriate section before selecting a response.

Materials & experimental systems

n/a	Involved in the study	
☐	☒	Antibodies
☐	☒	Eukaryotic cell lines
☒	☐	Palaeontology and archaeology
☒	☐	Animals and other organisms
☒	☐	Clinical data
☒	☐	Dual use research of concern

Methods

n/a	Involved in the study	
☒	☐	ChIP-seq
☒	☐	Flow cytometry
☒	☐	MRI-based neuroimaging

Antibodies

Antibodies used	Anti-PD-L1 antibody [EPR19759],ab213524, Abcam; GAPDH (14C10) Rabbit mAb, #2118, CST; Stat1 (D1K9Y) Rabbit mAb, #14994, CST; pStat1 (Tyr701) (D4A7) Rabbit mAb, #7649, CST; Anti-rabbit IgG, HRP-linked Antibody, #7074, CST; RIG-I Pathway Antibody Sampler Kit, #8348 CST; Innate Immunity Activation Antibody Sampler Kit, #52239 CST
Validation	www.abcam.com; www.cellsignal.com

Eukaryotic cell lines

Policy information about cell lines and Sex and Gender in Research

Cell line source(s)	KYSE-450, Japanese Collection of Research Bioresources Cell Bank, National Institutes of Biomedical Innovation; A549-Dual cells, Invitrogen (Carlsbad, CA, USA)
Authentication	mRNA-seq experiments shows that same gene expression profiles as the other published data.
Mycoplasma contamination	The cell lines are negative for cytoplasma contamination.
Commonly misidentified lines (See ICLAC register)	No commonly misidentified cell lines were used.
